# The CpG dinucleotide content of the HIV-1 envelope gene may predict disease progression

**DOI:** 10.1038/s41598-017-08716-1

**Published:** 2017-08-15

**Authors:** Mishi Kaushal Wasson, Jayanta Borkakoti, Amit Kumar, Banhi Biswas, Perumal Vivekanandan

**Affiliations:** Kusuma School of Biological Sciences, Indian Institute of Technology, Delhi, 110016 India

## Abstract

The clinical course of HIV-1 varies greatly among infected individuals. Despite extensive research, virus factors associated with slow-progression remain poorly understood. Identification of unique HIV-1 genomic signatures linked to slow-progression remains elusive. We investigated CpG dinucleotide content in HIV-1 envelope gene as a potential virus factor in disease progression. We analysed 1808 HIV-1 envelope gene sequences from three independent longitudinal studies; this included 1280 sequences from twelve typical-progressors and 528 sequences from six slow-progressors. Relative abundance of CpG dinucleotides and relative synonymous codon usage (RSCU) for CpG-containing codons among HIV-1 envelope gene sequences from typical-progressors and slow-progressors were analysed. HIV-1 envelope gene sequences from slow-progressors have high-CpG dinucleotide content and increased number of CpG-containing codons as compared to typical-progressors. Our findings suggest that observed differences in CpG-content between typical-progressors and slow-progressors is not explained by differences in the mononucleotide content. Our results also highlight that the high-CpG content in HIV-1 envelope gene from slow-progressors is observed immediately after seroconversion. Thus CpG dinucleotide content of HIV-1 envelope gene is a potential virus-related factor that is linked to disease progression. The CpG dinucleotide content of HIV-1 envelope gene may help predict HIV-1 disease progression at early stages after seroconversion.

## Introduction

Infection with Human Immunodeficiency virus 1 (HIV-1) is a major global problem. A small proportion of HIV-1 seropositive individuals are asymptomatic for several years and are able to maintain their CD4^+^ T-cell counts with low virus loads. Based on viral load, CD4+ counts and clinical symptoms, HIV-1 infected individuals are classified as typical-progressors, slow-progressors and virus controllers. The slow-progressor phenotype includes slow-progressors, late progressors, long-term non progressors (LTNP), long-term survivors and non-progressors^1–3^.

Slow progression of HIV-1-related disease has been linked to host-genetics, immunological factors and virus-related factors^4, 5^. Virus-factors including deletions in the long-terminal repeat and the *nef* gene^6^ and point mutations in specific genes^7^ have been reported in slow-progressors. While reduced virus fitness appears to be the unifying theme among slow-progressors, virus-related factors differ among this group of patients^8^. In other words, no known virus-related factor is consistently detected among slow-progressors. Understanding virus-related factors among slow-progressors remains an area of intense research owing to its importance in understanding the pathogenesis of HIV-1 and in developing a candidate vaccine^9^.

The relative abundance of dinucleotides in virus genomes provides important clues on virus pathogenesis and virus evolution^10, 11^. The CpG dinucleotide content of viruses has received much attention recently^12, 13^ and it has implications on (a) virus evolution^10, 11^ (b) virus replication^14^ (c) virus pathogenesis^15^ and (d) evasion of immune response^16^. CpG dinucleotides are under-represented in HIV-1 genomes^17, 18^. HIV-1 genomes are CpG depleted and the main source of CpG depletion is postulated to be host-induced methylation^19, 20^. Studies on virus-related factors in HIV-1 disease progression have not investigated the relative abundance of CpG dinucleotides. We hypothesized that the relative abundance of CpG dinucleotides in HIV-1 genomes influences disease progression.

To test this hypothesis we studied HIV-1 *env* gene sequences from typical-progressors and slow-progressors submitted previously^2, 21^. Our findings highlight (a) the association between CpG content of the HIV-1 *env* gene and disease progression and (b) the potential use of CpG-content in the HIV-1 *env* gene for predicting disease progression in the early stages of HIV-1 infection. In addition, this work provides a novel perspective on the pathogenesis and disease progression in HIV-1 infected individuals.

## Results and Discussion

### Increased CpG-content of HIV-1 *env* gene is linked to slow disease progression

The differences in the relative abundance of dinucleotides between typical-progressors and slow-progressors for datasets 1, 2 and 3 are shown in Supplementary Figure 1a,b and c respectively. The median difference in the dinucleotide content between the typical-progressors and slow-progressors was <17% for all dinucleotides except for the CpG dinucleotide. The average CpG dinucleotide content in slow-progressors is at least 30% higher as compared to that in typical-progressors in the three datasets (Supplementary Figure 1a,b and c). Box plots comparing the distribution of CpG_O/E_ ratios in typical-progressors and slow-progressors in datasets 1, 2 and 3 are shown in Figure﻿s 1a,b and c respectively. The median CpG_O/E_ values in slow-progressors were significantly higher than that in the typical-progressors (Figure﻿s 1a,b,c and Supplementary Figure 2a,b,c and d). Bar graphs in Figure﻿s 2a,b and c indicate the proportion of clones from typical-progressors and slow-progressors within a given CpG_O/E_ range. Figure 2a,b and c clearly show that a higher proportion of clones from typical-progressors have CpG_O/E_ values of 0.25 or less, while the majority of clones from slow-progressors had CpG_O/E_ values of 0.25 or more. In addition, box plots showing the distribution of CpG_O/E_ values for each patient studied from all the three datasets is shown in Supplementary Figure 3.

**Figure 1:**
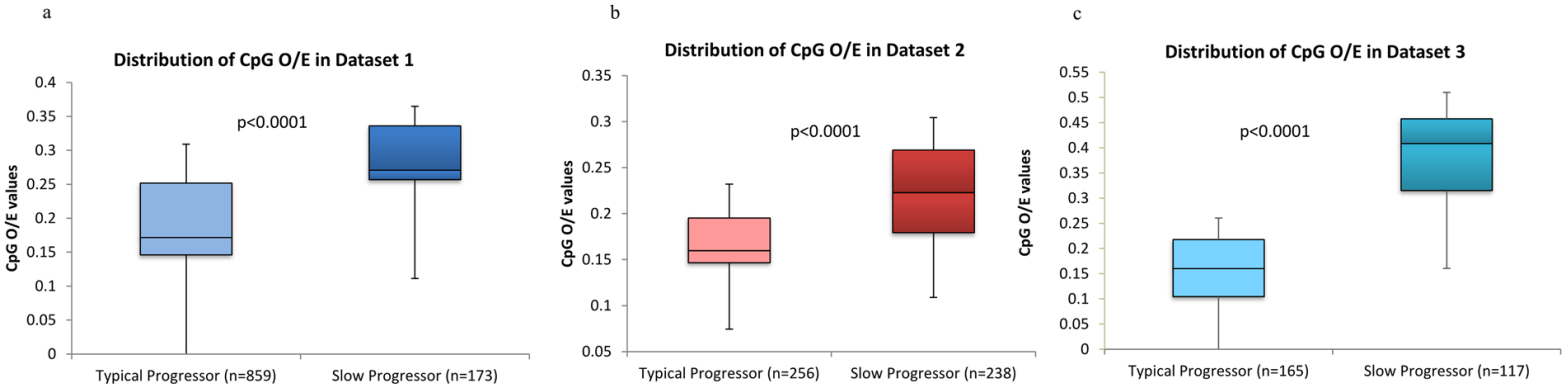
Higher CpG dinucleotide content in slow-progressors than in typical-progressors. Box plots comparing CpG_O/E_ ratios in typical-progressors and slow-progressors from (**a**) dataset 1 (**b**) dataset 2 and (**c**) dataset 3. P values were estimated using the Mann-Whitney test.

**Figure 2:**
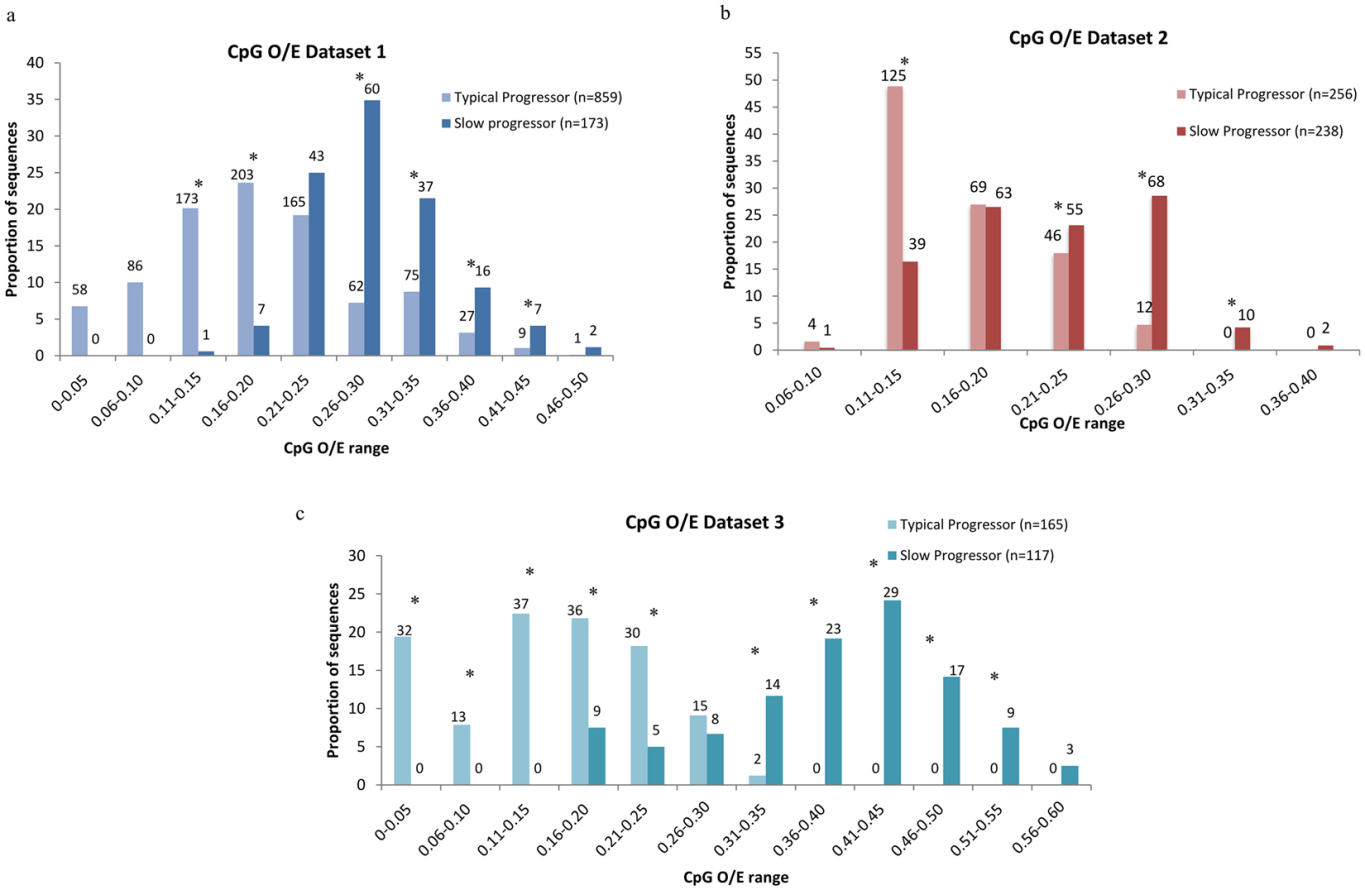
Distribution of CpG dinucleotide content in typical-progressors and slow-progressors. Bar graph showing the distribution of CpG_O/E_ ratios in typical-progressors and slow-progressors from (**a**) dataset 1 (**b**) dataset 2 and (**c**) dataset 3. (“*”Indicates χ^2^ test square values < 0·05). The numbers above the bars indicate the number of clones analysed.

Our findings suggest a potential role for CpG-dinucleotide content as a yet unknown virus factor in the pathogenesis of HIV-1 infection. Recent reports using synthetic virus constructs suggest a link between high CpG-content and attenuation of dengue virus^22^ and echovirus 7^23^. Although depletion of CpG-dinucleotides in the HIV-1 genome has been known for decades^18^, its influence, if any, on the pathogenesis of HIV-1 has not been investigated. A recent report indicates that influenza A virus mutants with high-CpG content are associated with loss of pathogenicity in mice; in addition, the high-CpG strains were associated with enhanced immune response^15^. Similarly, in another study recoded (altering the nucleotides with the codon without altering the amino acid) dengue virus 2 with high-CpG content was found to attenuate pathogenicity while inducing high levels of protective antibody response^22^. In addition, toll-like receptor 9 (TLR9) contributes to antiviral immunity by recognizing unmethylated CpG dinucleotides in virus genomes^24^. It is therefore possible that virus genomes with higher CpG content may elicit a better immune response from the host. Our finding of high-CpG content in the HIV-1 envelope gene from slow-progressors, supports a potential role for CpG-content as a modulator of pathogenesis and disease outcome. In addition, high CpG content of the HIV-1 envelope gene (C2-V5 region) represent a potential virus factor among slow-progressors. Prospective cohort studies may help further evaluate the clinical utility of CpG content in the prediction of HIV-1 disease progression. Methylation of CpGs in virus genomes can lead to the loss of CpG dinucleotides in virus genomes^12, 13^. Studies on CpG methylation of the HIV-1 proviral genomes are focussed on the LTR-region^25, 26^. Extensive methylation of CpGs in the envelope gene of a non-progressor has been reported^25^. It is therefore difficult to comment on the possible association between the methylation of HIV-1 proviral DNA and the observed differences in the CpG content between typical-progressors and slow-progressors.

### Increased CpG-content in slow-progressors is not linked to the differences in mononucleotide composition

We randomized all the sequences studied by shuffling them without changing the mononucleotide frequency (n = 1808; as described in the methods section) and then re-analyzed the CpG content. Interestingly, the CpG content of the HIV-1 sequences in the typical-progressors and slow-progressors were comparable after randomization (Figure﻿s 3a,b and c). This finding vindicates that the high CpG-content observed in the slow-progressors is not influenced by the differences, if any, in the constituent mononucleotides.

**Figure 3:**
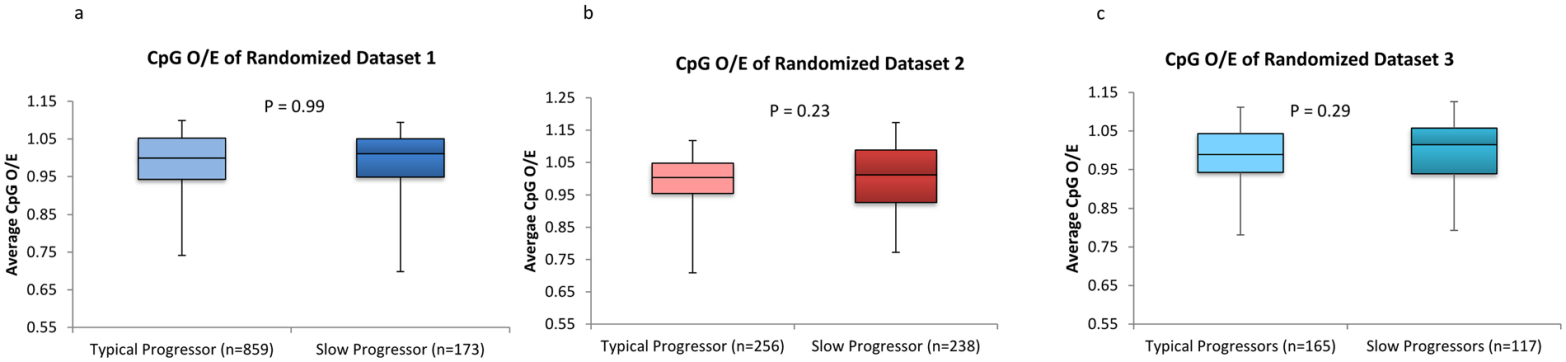
Analysis of CpG-dinucleotide content after randomization of sequences suggest that the differences in CpG-content between typical-progressors and slow-progressors is not explained by the differences in mononucleotide composition. Sequences in dataset 1, dataset 2 and dataset 3 were randomized without changing the overall mononucleotide content (as described in the methods section). CpG_O/E_ ratios were calculated for the randomized sequences. Box plots comparing CpG_O/E_ ratios in slow-progressors and typical-progressors after randomization of sequences in (**a**) dataset 1 (**b**) in dataset 2 and (**c**) in dataset 3. P values were estimated using the Mann-Whitney test.

### Increased usage of CpG-containing codons is linked to slow progression of HIV-1 disease

We investigated the differences in RSCU values of codons within the HIV-1 envelope gene (C2-V5 region). We then analysed the RSCU values in the context of the dinucleotide content within the codons^13^. The differences in the RSCU values of different dinucleotide containing codons between typical-progressors and slow-progressors for datasets 1, 2 and 3 are shown in Supplementary Figures 4a,b and c respectively. The median fold change in the RSCU values of CpG-dinucleotide containing codons between typical-progressors and slow-progressors was >18% (Supplementary Figure 4a,b and c). The median RSCU values for CpG-containing codons were significantly higher in the slow-progressors as compared to that in the typical-progressors in all the three datasets (Figure﻿s 4a,b and c). In other words, CpG-containing synonymous codons were more frequently used in HIV-1 sequences from the slow-progressors than that from typical-progressors. In HIV-1 infected slow-progressors/long-term non-progressors the virus loads are typically much lower than that seen in typical-progressors^1, 4^. In addition, studies on HIV-1 disease progression indicate the existence of a stronger and a more broadly neutralizing immune response among slow-progressors/long-term non-progressors as compared to typical-progressors^27^. Recent reports indicate that increasing the number of CpG-containing codons in influenza A virus^15^ echovirus 7^23^ and dengue virus type-2^22^ result in low levels of replication. Importantly, the increased number of CpG-containing codons enhanced the immune response in mice to influenza A virus^15^. We speculate that the increased numbers of CpG-containing codons in the HIV-1 sequences from slow-progressors may also be linked to the low levels of virus replication and a better immune response.

**Figure 4:**
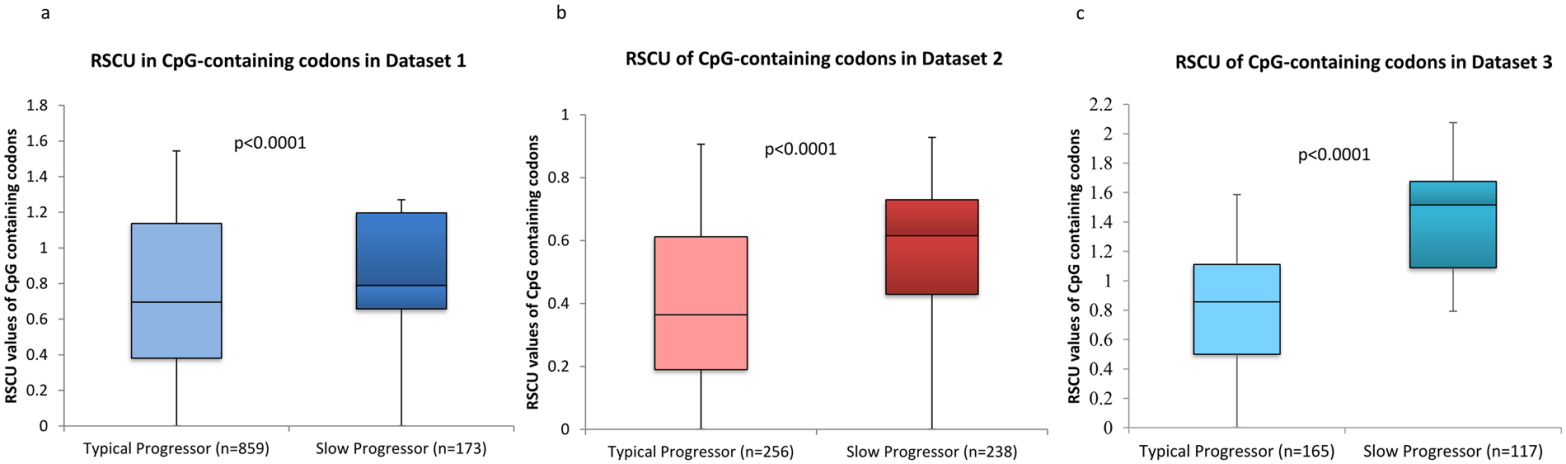
Higher RSCU values for CpG-containing codons in slow-progressors than in typical-progressors. Box plots comparing RSCU values of CpG-containing codons in typical-progressors and slow-progressors from (**a**) dataset 1 (**b**) dataset 2 and (**c**) dataset 3. P values were estimated using the Mann-Whitney test.

The C2-V5 region of the HIV-1 envelope gene analysed in this study is an important target for designing vaccines^28^ and entry inhibitors^29^. This region has been reported to elicit strong neutralizing antibody responses in HIV-1 slow-progressors/long-term non-progressors (4,27,28). Research on HIV-1 vaccines and antivirals have focused on differences in the epitopes and their structure and conformation. To our knowledge, the differences in CpG content in the genome or within the codons have not been considered for HIV-1 vaccine/anti-viral design. Our findings shed new light on the potential importance of CpG dinucleotide content in the designing of HIV-1 vaccines.

### Differences in the CpG-content between typical-progressors and slow-progressors are evident immediately after seroconversion

**Figure 5:**
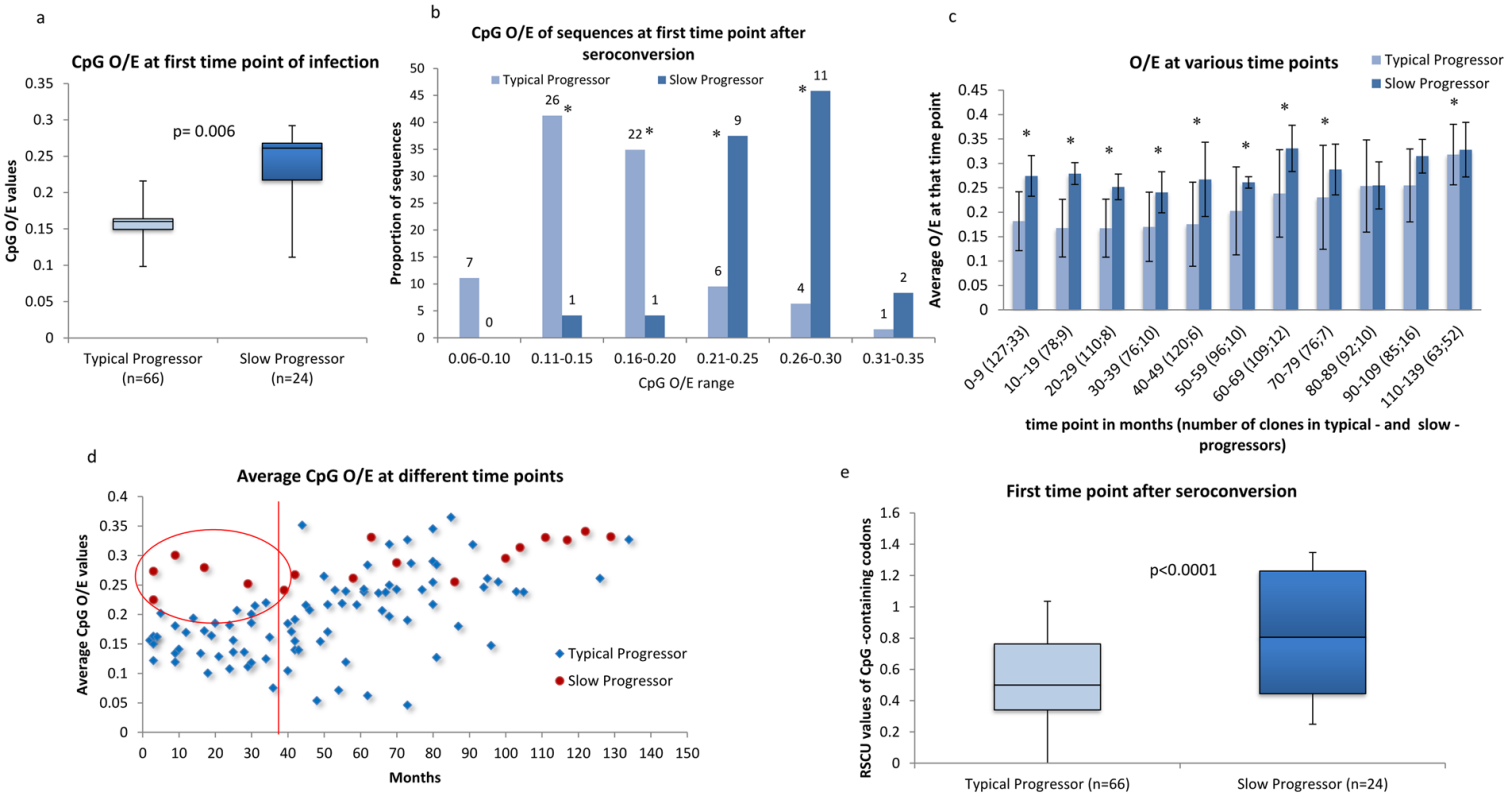
Comparison of CpG-content between typical-progressors and slow-progressors in the first time point (within 5 months) after seroconversion and during follow-up from dataset 1. (**a**) Box plots comparing CpG_O/E_ ratios between typical-progressors and slow-progressors at the first time point after seroconversion (i.e. within 5 months of seroconversion) in dataset 1. The P value was estimated using Mann-Whitney test. (**b**) Bar graphs showing the distribution of CpG_O/E_ ratios in typical-progressors and slow-progressors at the first time point after seroconversion from dataset 1. The number above the bars represents the number of clones analyzed (“*”χ^2^ test p value < 0·05) (**c**) Bar graphs illustrating that the average CpG_O/E_ ratios in sequences from slow-progressors are higher than that from typical-progressors at most time points during the entire follow-up period. (“*”χ^2^ test p value < 0·05), the error bars represent standard deviation. The number of clones analyzed is given in parenthesis (**d**) A scatter plot illustrating CpG_O/E_ ratios in sequences from slow-progressors are not over-lapping with that from the typical-progressors in the first 3 years of after seroconversion (**e**) Box plots comparing RSCU values of CpG-containing codons in typical-progressors and slow-progressors at the first time point after seroconversion (within 5 months of seroconversion) in dataset 1. The P value was estimated using the Mann-Whitney test.

To ascertain, if the differences in the relative abundance of CpG dinucleotides and the RSCU values of CpG-containing codons are significantly different between typical-progressors and slow-progressors in the early stages of HIV-1 infection (i.e. within a few months of seroconversion), we studied dataset 1, as all HIV-1 infected patients in this study were sampled within the first five months after seroconversion. Sequences from dataset 2 and 3 were excluded for this analysis as most patients in dataset 2 and 3 were sampled at about 7 years or later after their HIV-1 seropositive result. Interestingly, the relative abundance of CpG dinucleotides in the first-time point after seroconversion (i.e within 0–5 months of seroconversion in dataset 1) was significantly higher among slow-progressors as compared to that in typical-progressors (Figure﻿s 5a,b and Supplementary Figure 5). In addition, at the first-time point after seroconversion 95% of sequences from slow-progressors had CpG_O/E_ ratios ≥0·20; while only 16% of sequences from typical-progressors had CpG_O/E_ ratios ≥0·20 (Figure﻿s 5c and d). Furthermore, at the first-time point after seroconversion HIV-1 sequences from slow-progressors had higher RSCU values for CpG-containing codons as compared to that from typical-progressors (Figure 5e). Taken together, these findings clearly indicate that the high-CpG content in slow-progressors is seen at the very early stages of HIV-1 infection. Furthermore, this finding also suggests that the high-CpG content in slow-progressors is a potential virus-related factor in HIV-1 disease progression. In other words, the high-CpG content in slow-progressors at the very early stages of HIV-1 infection argues against a gradual host-mediated selection of quasi-species with a high-CpG content in this group of patients. This finding also suggests that the CpG-content of the HIV-1 envelope gene (C2-V5) at the time of infection may influence HIV-1-related disease progression.

We then compared the average CpG_O/E_ ratios between typical-progressors and slow-progressors at various time-points after seroconversion; this was done on dataset 1 as patients were sampled at regular intervals after seroconversion. Of note, the average CpG_O/E_ ratios among slow-progressors were absolutely non-overlapping with that in typical-progressors up to the first three years (Figure 5d). In addition, at most time-points post-seroconversion (0–12 years), the relative abundance of CpG dinucleotides was higher among slow-progressors as compared to typical-progressors (Figure 5c). Predicting HIV-1-related disease progression in the early stages of HIV-1 infection using virus loads and/or CD4 cell counts remains a challenge^1, 4, 30^. Sequential measurements of virus loads and CD4 cell counts for several years are required to predict the outcome of HIV-1-related disease progression^1, 30^. The high CpG-content among slow-progressors at the early stages of seroconversion in our study indicates that CpG-content of the HIV-1 envelope gene can be potentially used as a marker of HIV-1 disease progression immediately after seroconversion. APOBECs are associated with G to A hypermutation. While several hotspots or nucleotide sequence preferences have been identified for APOBEC-mediated editing^31^, in terms of dinucleotide content, APOBECs do not show any preferences^32^; nonetheless, a role for APOBECs in the evolution of CpG content cannot be ruled out.

While our study highlights the potential link between HIV-1 CpG content and disease progression, it has certain limitations: (a) we could only use partial envelope gene sequences as full length envelope gene sequences were not available from the three cohorts. (b) Some patients were on anti-viral therapy which could potentially impact virus evolution. (c) We are not able to elicit the specific underlying mechanism leading to CpG evolution. (d) Each of the three longitudinal cohorts (from which we analysed sequences) used unique criteria for identifying the slow progressors. (e) Most of the sequences analysed in our study are from HIV-1subtype B, while subtype C is the most predominant subtype.

Understanding virus-factors in HIV-1-related disease progression remains elusive. In this study we have identified CpG content of the HIV-1 envelope gene as a potential virus factor that is linked to HIV-1 disease progression. High CpG-content and increased usage of CpG-containing codons were consistent features of the HIV-1 envelope gene from slow-progressors; this suggests a potential role for CpG content in the pathogenesis of HIV-1. Differences in CpG content between typical-progressors and slow-progressors are evident immediately after seroconversion. We believe that the identification of CpG content of the HIV-1 envelope gene as a potential virus factor in the pathogenesis and disease progression in HIV-1 infected individuals will facilitate a plethora of studies.

## Materials and Methods

### Retrieval of sequences and HIV-1 *env* gene analysed

We chose to study the *env* gene of HIV-1 because (a) the residues in the envelope protein of HIV-1 have been reported to be important for virus escape and selection^33^ (b) a recent report on dengue virus suggests that the CpG content of the virus envelope gene is linked to pathogenesis and immune response^22^. We analysed a total of 1808 HIV-1 *env* sequences reported in three independent longitudinal studies. This includes 1032 nucleotide sequences from the C2-V5 region of the HIV-1 envelope gene from nine patients consisting of seven typical-progressors and two slow-progressors phenotypes submitted by Shankarappa *et al*.^2^; the sequences from this cohort are subsequently referred to as dataset 1. In this study all participants were recruited within the first five months of becoming infected with HIV-1 and were followed-up semi-annually for 6–12 years. The accession numbers of sequences in dataset 1 include AF137629 to AF138163, AF138166 to AF138263, and AF138305 to AF138703. We analysed 494 sequences from the C2-V5 region from six patients (three typical-progressors and three slow- progressors) submitted by Bagnarelli *et al*.^21^; the sequences from this cohort are subsequently referred to as dataset 2. In dataset 2, five of the six patients were followed-up from about seven years to ten years after the first seropositive result; one patient was followed-up from one year to four years after the first seropositive result. The accession numbers of sequences in dataset 2 include AF105432 to AF105680 and AF105717 to AF105961. In addition, 282 sequences from C2-V5 region in three patients (two typical-progressors and one slow-progressor) submitted by Bello *et al*.^34^ were analysed; sequences from this cohort are subsequently referred to as dataset 3. In dataset 3, there were two typical-progressors and two slow-progressors, who were followed-up for a period of 6 to 7 years (between 2 to 16 years after seroconversion). The accession numbers of sequences in dataset 3 included AY497931 to AY498168, AY498227 to AY498346, AY501319, AY501321, AY501332, AY501333, EF118762 to EF118806 and EU643991 to EU643999. The details of CD4+ T cell count, virus loads and antiviral therapy for patients in all the three data sets are given in Supplementary Table 1. Additional details including the country in which the three studies were carried out, HIV-1 subtype information, time-points of sampling and number of clones sequenced at each time point along with the accession numbers as provided in the three original papers^2, 21, 34^ are summarized in Supplementary Table 2.

A small subset of slow-progressors with very low virus loads for several years without antiviral therapy are recognized as elite controllers^1, 35^. Significant differences in the underlying mechanisms between slow-progressors and virus controllers are recognized in, virus and host factors^36, 37^. Since all the three papers from which sequences were analysed for this study were published before the time period when elite controllers were recognized as a clinical entity that is distinct from slow-progressors, we assessed if any of the seven slow-progressors from the three data sets would qualify as elite controllers. There are no standard criteria used to describe elite controllers^1^; we used the following criteria to identify elite controllers, if any, from the three datasets: virus loads of <400 copies/mL without antiviral therapy for at least 10 years after seroconversion^1, 35^. We identified 1 of the slow-progressors from dataset 3 who qualified as an elite controller; this patient (patient 45) had median virus loads of less than 400 copies /mL for over 17 years after seroconversion without antiviral therapy. Therefore, sequences (n = 132) from this elite controller were excluded from further analysis. In addition, two sequences which were much smaller in length (<250 bp) compared to other sequences analysed (>600 bp) were excluded.

### Calculation of dinucleotide frequencies

The relative abundance of all dinucleotides including the CpG dinucleotide was calculated as observed/expected ratios (O/E ratios) as described previously^12^. For example, the O/E ratio for dinucleotide XpY is calculated using the formula: [XpY_O/E_ = f(XY)/f(X)f(Y)]G. Here, f(XY) is the observed frequency of the dinucleotide and f(X)f(Y) is the product of the mononucleotides that constitute the dinucleotide and G is the length of the genome^12^.

### Calculation of RSCU values

Relative synonymous codon usage (RSCU) is the ratio of the observed frequency of codons to the expected frequency under equal synonymous codon usage. RSCU values indicate codon usage bias. RSCU values for the sequences were calculated using an online tool http://genomes.urv.es/CAIcal ^38^. A RSCU value greater than 1 implies a positive codon bias whereas RSCU values of less than 1 imply negative codon usage. RSCU is calculated as: RSCU _**i**_ = X_i_/1/n $\sum _{i=1}^{n}X{\rm{i}}$, where n = number of synonymous codons, (1 ≤ *n* ≤ 6) for the amino acid under study, *X*_i_ = number of occurrences of codon *i*.

### Randomization of sequences

To investigate if the differences in CpG_O/E_ ratios between slow-progressors and typical-progressors were due to inherent difference in mononucleotide composition we first randomized the sequences studied. Briefly, each sequence (n = 1808) was randomized (or shuffled) five times without changing the mononucleotide composition using an online randomization tool (http://www.bioinformatics.org/sms2/shuffle_dna.html)^39^. Each sequence was analysed for its CpG_O/E_ content and the average of five randomized CpG_O/E_ values was calculated. We then analysed the CpG_O/E_ ratios in the randomized sequences from slow-progressors and typical-progressors. If the observed differences in CpG_O/E_ ratios between slow-progressors and typical-progressors are preserved after randomization, it will suggest that the inherent differences in the mononucleotide composition contributes to the observed differences in the CpG content. In contrast, if the CpG_O/E_ ratios in typical-progressors and slow-progressors are comparable after randomization, it would indicate that the observed differences in CpG content are independent of differences in mononucleotide composition.

### Statistical analysis

Data were analysed using Mann Whitney test and χ^2^ test, as appropriate. Box plots and frequency distribution bar diagrams were created using Microsoft Excel 2013. P values < 0.05 were considered significant.

## Electronic supplementary material

Supplementary Files

## References

[1] Gurdasani D (2014). A systematic review of definitions of extreme phenotypes of HIV control and progression. AIDS.

[2] Shankarappa R (1999). Consistent viral evolutionary changes associated with the progression of human immunodeficiency virus type 1 infection. J. Virol..

[3] Crotti A (2006). Nef Alleles from Human Immunodeficiency Virus Type 1-Infected Long-Term-Nonprogressor Hemophiliacs with or without Late Disease Progression Are Defective in Enhancing Virus Replication and CD4 Down-Regulation. J. Virol..

[4] Poropatich, K., Sullivan, D. J. & Sullivan, D. J. Human immunodeficiency virus type 1 long-term non-progressors: the viral, genetic and immunological basis for disease non-progression. *J. Gen Virol.* 247–268, doi:10.1099/vir.0.027102-0 (2016). 10.1099/vir.0.027102-0 21106806

[5] Zaunders J, Van Bockel D (2013). Innate and adaptive immunity in long-term non-progression in HIV disease. Front. Immunol..

[6] Churchill MJ (2006). Longitudinal Analysis of Human Immunodeficiency Virus Type 1 nef/Long Terminal Repeat Sequences in a Cohort of Long-Term Survivors Infected from a Single Source Longitudinal Analysis of Human Immunodeficiency Virus Type 1 nef/Long Terminal Repeat Sequ. J. Virol..

[7] Cruz NV, Amorim R, Oliveira FE, Speranza FA (2013). Mutations in the nef and vif Genes Associated With Progression to AIDS in Elite Controller and Slow-Progressor Patients. J. Med. Virol..

[8] Weber J (2017). Impaired human immunodeficiency virus type 1 replicative fitness in atypical viremic non-progressor individuals. AIDS Res. Ther..

[9] Wang B (2013). Viral factors in non-progression. Front. Immunol..

[10] Cheng, X. *et al*. CpG Usage in RNA Viruses: Data and Hypotheses. *PLoS One* **8** (2013). 10.1371/journal.pone.0074109 PMC3781069 24086312

[11] Greenbaum BD, Levine AJ, Bhanot G, Rabadan R (2008). Patterns of evolution and host gene mimicry in influenza and other RNA viruses. PLoS Pathog..

[12] Upadhyay, M. *et al*. CpG Dinucleotide Frequencies Reveal the Role of Host Methylation Capabilities in Parvovirus Evolution. **87**, 13816–13824 (2013). 10.1128/JVI.02515-13 PMC3838256 24109231

[13] Upadhyay, M. & Vivekanandan, P. Depletion of CpG Dinucleotides in Papillomaviruses and Polyomaviruses: A Role for Divergent Evolutionary Pressures. 1–16, doi:10.1371/journal.pone.0142368 (2015). 10.1371/journal.pone.0142368 PMC4636234 26544572

[14] Burns CC (2009). Genetic inactivation of poliovirus infectivity by increasing the frequencies of CpG and UpA dinucleotides within and across synonymous capsid region codons. J. Virol..

[15] Gaunt E (2016). Elevation of CpG frequencies in influenza a genome attenuates pathogenicity but enhances host response to infection. Elife.

[16] Jimenez-Baranda S (2011). Oligonucleotide motifs that disappear during the evolution of influenza virus in humans increase alpha interferon secretion by plasmacytoid dendritic cells. J Virol.

[17] Shpaer, E. G. & Mullins, J. I. Selection against CpG dinucleotides in lentiviral genes: a possible role of methylation in regulation of viral expression. **18**, 5793–5797 (1990). 10.1093/nar/18.19.5793 PMC332316 2170945

[18] Karlin, S., Doerfler, W. & Cardon, L. R. Why Is CpG Suppressed in the Genomes of Virtually All Small Eukaryotic Viruses but Not in Those of Large Eukaryotic Viruses? **68**, 2889–2897 (1994). 10.1128/jvi.68.5.2889-2897.1994 PMC236777 8151759

[19] van der Kuyl AC, Berkhout B (2012). The biased nucleotide composition of the HIV genome: a constant factor in a highly variable virus. Retrovirology.

[20] Alinejad-Rokny H, Anwar F, Waters SA, Davenport MP, Ebrahimi D (2016). Source of CpG Depletion in the HIV-1 Genome. Mol. Biol. Evol..

[21] Bagnarelli P (1999). Host-specific modulation of the selective constraints driving human immunodeficiency virus type 1 env gene evolution. J. Virol..

[22] Shen SH (2015). Large-scale recoding of an arbovirus genome to rebalance its insect versus mammalian preference. Proc. Natl. Acad. Sci. USA.

[23] Atkinson NJ, Witteveldt J, Evans DJ, Simmonds P (2014). The influence of CpG and UpA dinucleotide frequencies on RNA virus replication and characterization of the innate cellular pathways underlying virus attenuation and enhanced replication. Nucleic Acids Res..

[24] Guggemoos S (2008). TLR9 contributes to antiviral immunity during gammaherpesvirus infection. J. Immunol..

[25] Weber S (2014). Epigenetic analysis of HIV-1 proviral genomes from infected individuals: Predominance of unmethylated CpG’s. Virology.

[26] Palacios JA (2012). Long-term nonprogressor and elite controller patients who control viremia have a higher percentage of methylation in their HIV-1 proviral promoters than aviremic patients receiving highly active antiretroviral therapy. J. Virol..

[27] Sreepian A (2004). Conserved neutralizing epitopes of HIV type 1 CRF01_AE against primary isolates in long-term nonprogressors. AIDS Res. Hum. Retroviruses.

[28] Zolla-Pazner S, Cardozo T (2010). Structure-function relationships of HIV-1 envelope sequence-variable regions refocus vaccine design. Nat. Rev. Immunol..

[29] Caffrey M (2011). HIV envelope: Challenges and opportunities for development of entry inhibitors. Trends Microbiol..

[30] Langford SE, Ananworanich J, Cooper DA (2007). Predictors of disease progression in HIV infection: a review. AIDS Res. Ther..

[31] Chen, J. *et al*. The preferred nucleotide contexts of the AID/APOBEC cytidine deaminases have differential effects when mutating retrotransposon and virus sequences compared to host genes. *PLOS Computational Biology* **13** (2017). 10.1371/journal.pcbi.1005471 PMC5391955 28362825

[32] Deforche K (2007). Estimating the relative contribution of dNTP pool imbalance and APOBEC3G/3F editing to HIV evolution *in vivo*. J. Comput. Biol..

[33] Mahalanabis M (2009). Continuous Viral Escape and Selection by Autologous Neutralizing Antibodies in Drug-Naive Human Immunodeficiency Virus Controllers. J. Virol..

[34] Bello G (2007). Lack of temporal structure in the short term HIV-1 evolution within asymptomatic naïve patients. Virology.

[35] Okulicz JF (2009). Clinical outcomes of elite controllers, viremic controllers, and long-term nonprogressors in the US Department of Defense HIV natural history study. J. Infect. Dis..

[36] Poropatich K, Sullivan DJ (2011). Human immunodeficiency virus type 1 long-term non-progressors: The viral, genetic and immunological basis for disease non-progression. J. Gen. Virol..

[37] Gaardbo, J. C., Hartling, H. J., Gerstoft, J. & Nielsen, S. D. Thirty years with HIV infection - Nonprogression is still puzzling: Lessons to be learned from controllers and long-term nonprogressors. *AIDS Res*. *Treat*. **2012** (2012). 10.1155/2012/161584 PMC3368166 22693657

[38] Puigbò P, Bravo IG, Garcia-Vallvé S (2008). E-CAI: a novel server to estimate an expected value of Codon Adaptation Index (eCAI). BMC Bioinformatics.

[39] P. S (2000). The Sequence Manipulation Suite: JavaScript programs for analyzing and formatting protein and DNA sequences. Biotechniques.

