# Supplementary material for: The CpG dinucleotide content of the HIV-1 envelope gene may predict disease progression
Source: Sci Rep. 2017 Aug 15;7:8162. doi: 10.1038/s41598-017-08716-1 (PMC5557942; doi:10.1038/s41598-017-08716-1)
Supplement: Supplementary file 1 — Supplementary Files [file 41598_2017_8716_MOESM1_ESM.pdf]

The CpG dinucleotide content of the HIV-1 envelope gene may predict disease progression

Mishi Kaushal Wasson [ǂ a] (PhD), Jayanta Borkakoti [ǂ a] (PhD), Amit Kumar, Banhi Biswas[a], Perumal Vivekanandan [*a] (PhD)

[a] Kusuma School of Biological Sciences, Indian Institute of Technology, Delhi, 110016

[ǂ] Mishi Kaushal Wasson and Jayanta Borkakoti contributed equally to this work

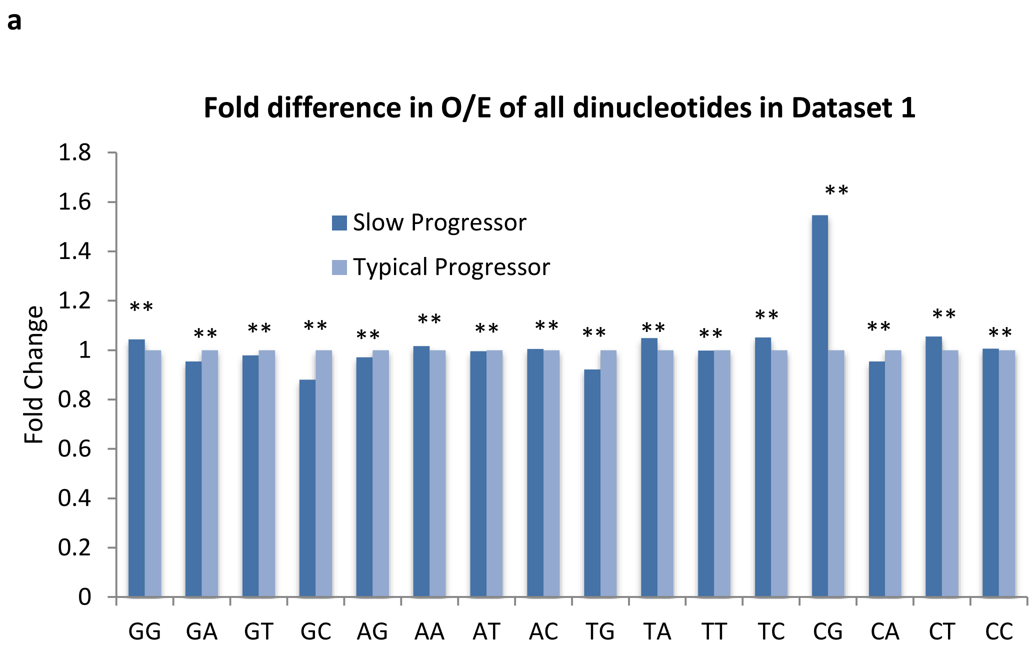

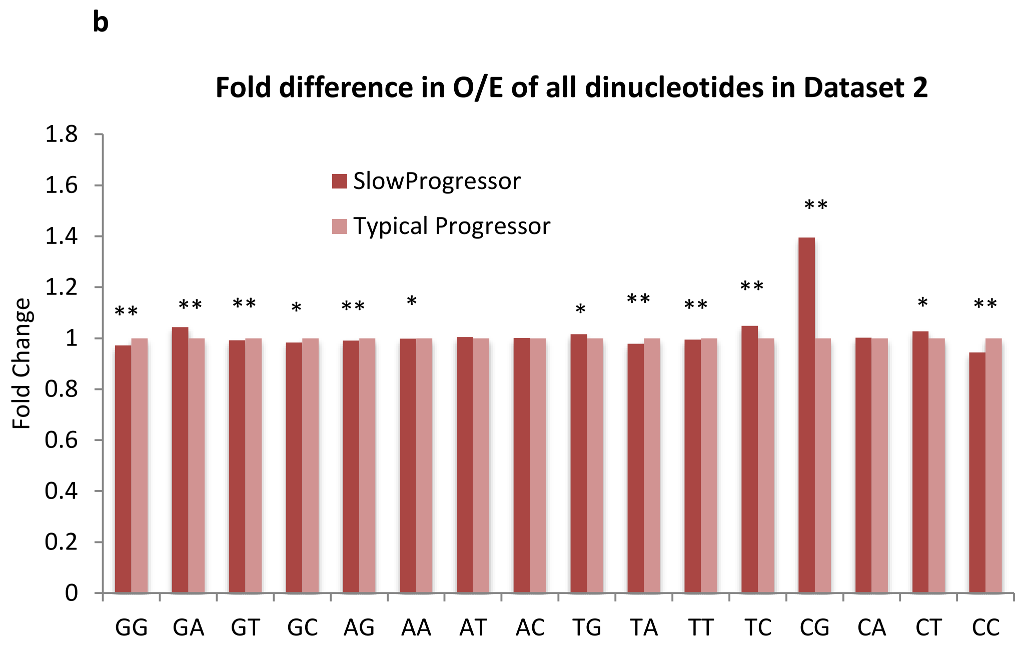

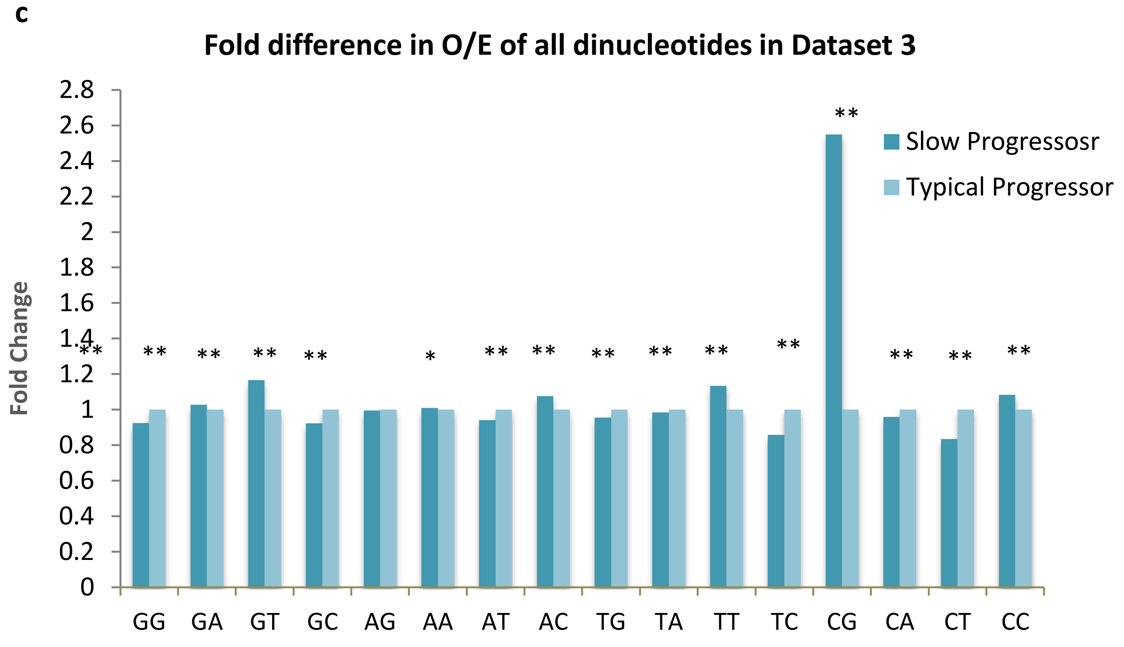

Supplementary Figure 1: Relative abundance of dinucleotides in typical-progressors and slow-progressors

The median fold change in the relative abundance ($_{O/E}$ ratios) of dinucleotides between typical-progressors and slow-progressors in (a) dataset 1 (b) in dataset 2 and (c) in dataset 3. P values were estimated using the Mann-Whitney test. ("*" signifies p<0.01 and "**" signifies p values < 0·0001).

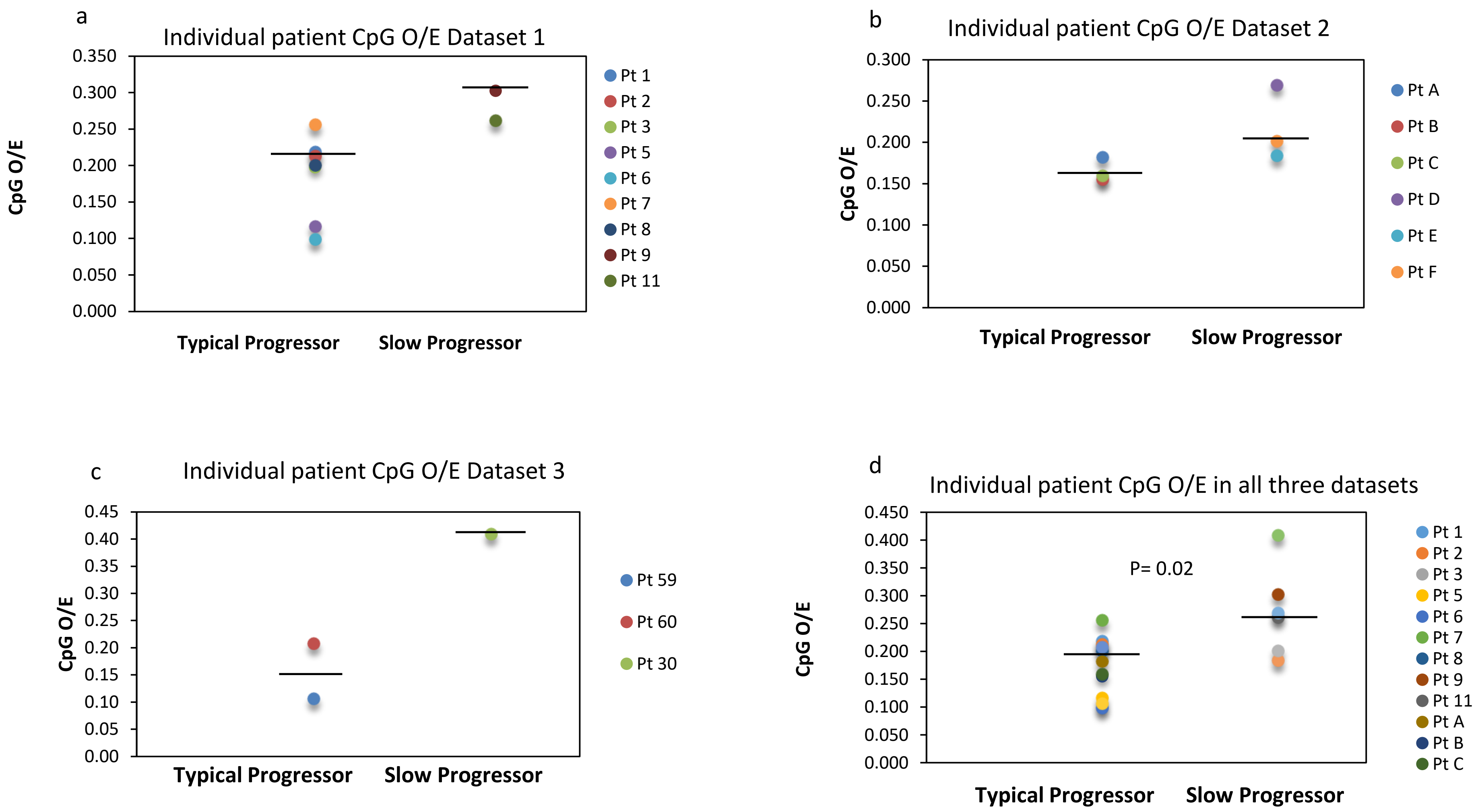

Supplementary Figure 2: Median CpG $_{O/E}$ values of individual patients

Median CpG $_{O/E}$ values of individual patients (all sequences) in (a) dataset 1, (b) dataset 2, (c) dataset 3 (d) all three dataset combined. P values were estimated using the Mann-Whitney test.

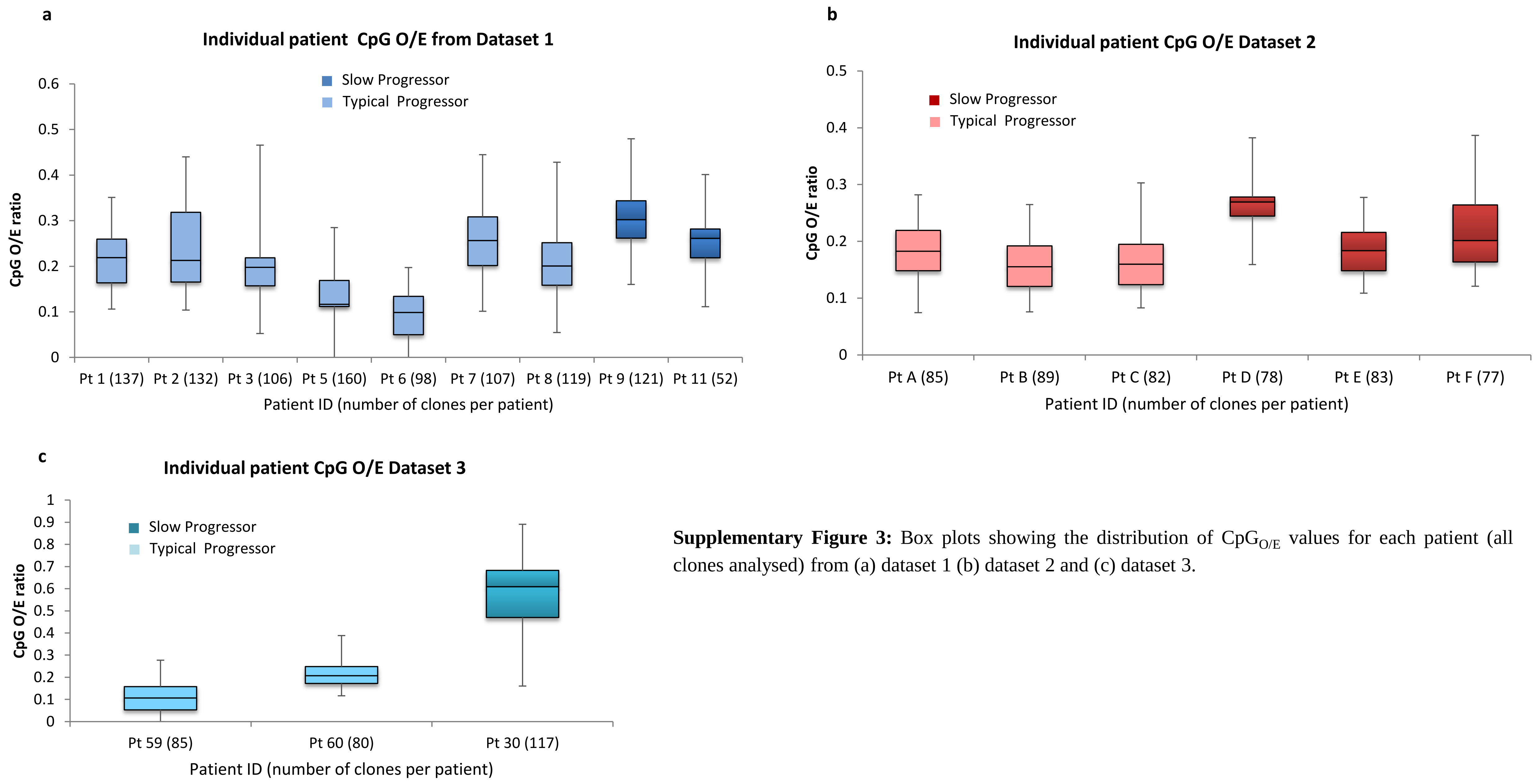

Supplementary Figure 3: Box plots showing the distribution of $CpG_{O/E}$ values for each patient (all clones analysed) from (a) dataset 1 (b) dataset 2 and (c) dataset 3.

a

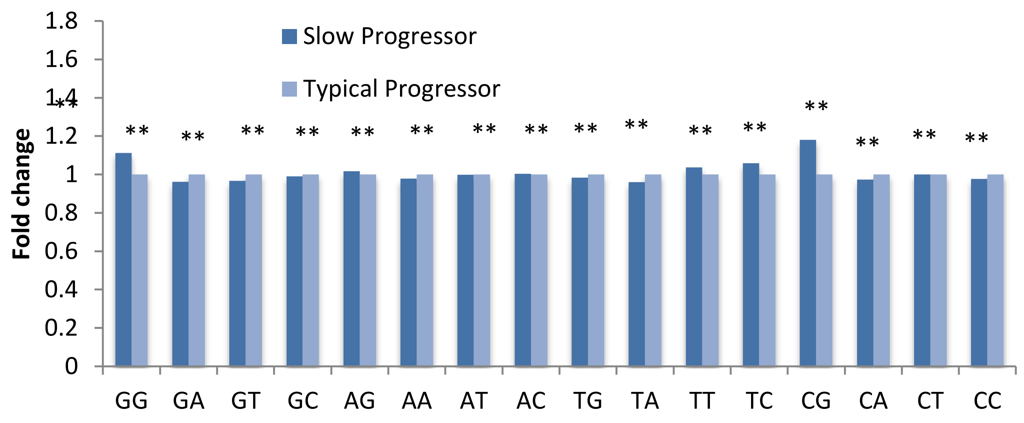

b

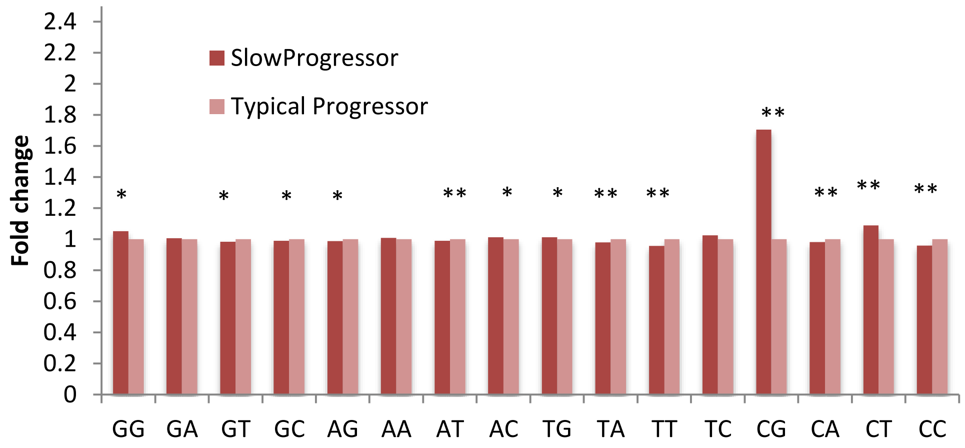

c

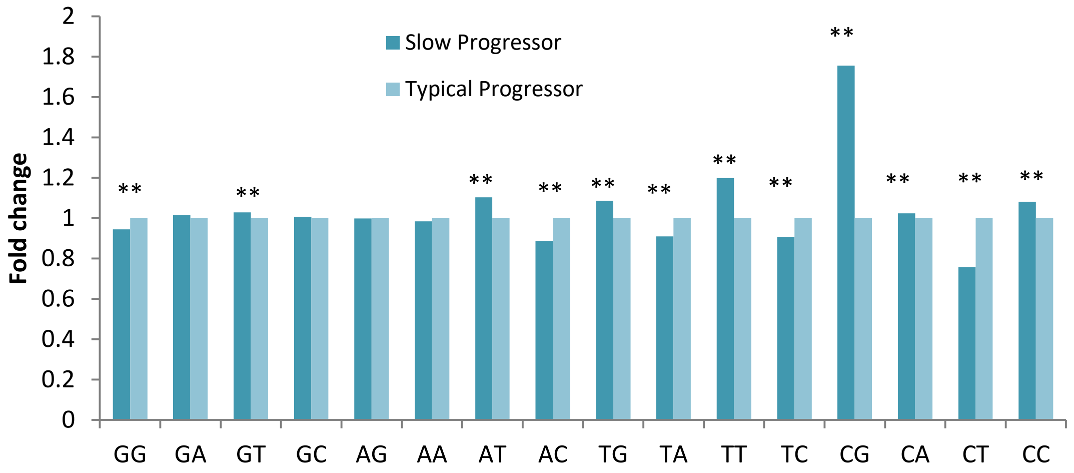

Supplementary Figure 4: Comparison of CpG-containing codon usage in slow-progressors and typical-progressors

Bar graph illustrating the median fold change in RSCU values of codons with different dinucleotides in slow-progressors as compared to typical-progressors in (a) dataset 1 (b) dataset 2 and (c) dataset 3. P values were estimated using the Mann-Whitney test ("* signifies p<0.01 and "**" signifies p values < 0·0001)

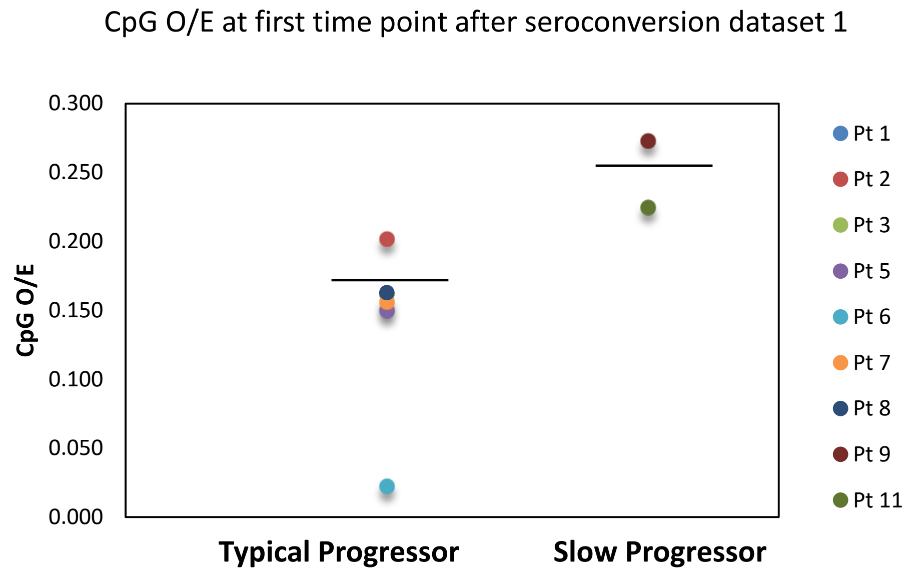

Supplementary Figure 5: Median CpG $_{O/E}$ values of individual patients (all sequences) in (a) dataset 1 at first time point after seroconversion in dataset 1

Supplementary Table 1: Details of CD4+ T cell count, virus loads and antiviral therapy for patients in all the three data sets

DATASET	$CD4^+$ T cells **	RNA VIRAL LOAD *(copies/ml)	ART	Sample	Classification by the auhtors of the original papers
**Dataset 1**					
Patient 1	< 600 cells /µl within 4 years	>4 log10 in ~ 2 years	Not documented	PBMCs	Typical-progressor
Patient 2	< 600 cells /µl within 7 years	>4 log10 within ~ 1 years	After 8 years	PBMCs	Typical-progressor
Patient 3	< 600 cells /µl within 5.5 years	>4 log10 within ~ 1 years	After 6 years	PBMCs	Typical-progressor
Patient 5	< 600 cells /µl within 4 years	>4 log10 within ~ 1 years	After 5 years	PBMCs	Typical-progressor
Patient 6	< 600 cells /µl within 1 year	>4 log10 within ~ 1 years	After 3 years	PBMCs	Typical-progressor
Patient 7	< 600 cells /µl within 1 year	>4 log10 within ~ 1 years	After 3 years	PBMCs	Typical-progressor
Patient 8	< 600 cells /µl within 1 year	>4 log10 within ~ 1 years	After 6 years	PBMCs	Typical-progressor
Patient 9	>600 cells/µl upto 8 years	>2.6 and <4 log10 till 8 years	After/Within 5 Years	PBMCs	Slow-progressor
Patient 11	>600 cells/µl upto10 years	>2.6 and <4 log10 till 8 years	No ART	PBMCs	Slow-progressor
**Dataset 2**					
Patient A	Declining CD4+ T cells with a mean loss of 125 CD4+ T cells/µl each year	>4 log10	No ART	PBMC s and plasma	Typical-progressor
Patient B	Declining CD4+ T cells with a mean loss of 125 CD4+ T cells/µl each year	>4 log10	No ART	PBMC s and plasma	Typical-progressor
Patient C	Declining CD4+ T cells with a mean loss of 125 CD4+ T cells/µl each year	>4 log10	No ART	PBMC s and plasma	Typical-progressor
Patient D	CD4+ T cells are maintained at > 600 cell/µl with a mean loss of 14 CD4+ T cells/µl each year	2.7 log10 in 10 years	No ART	PBMC s and plasma	Slow-progressor
Patient E	CD4+ T cells are maintained at > 600 cell/µl with a mean loss of 14 CD4+ T cells/µl each year	3.4 log10 in 7.5 years	No ART	PBMC s and plasma	Slow-progressor
Patient F	CD4+ T cells are maintained at > 600 cell/µl with a mean loss of 14 CD4+ T cells/µl each year	2.9 log10 in 10.5 hears	No ART	PBMC s and plasma	Slow-progressor
**Dataset 3**					
Patient 59	Declining $CD4^+$ levels (decline of > than 50 cells/µl each year)	~ 4 log10 within 5 years	No ART	PBMCs	Typical-progressor
Patient 60	Declining CD4+ levels (decline of > than 50 cells/µl each year)	~ 4 log10 within 2 years	No ART	PBMCs	Typical-progressor
Patient 30	CD4+ T cells are maintained at > 600 cell/µl (no significant decline in CD+T cells)	3.5 log10 in 9.5 years	No ART	PBMCs	Slow-progressor
Patient 45	CD4+ T cells are maintained at > 600 cell/µl (no significant decline in CD+T cells)	2.5 log10 in 17 years	No ART	PBMCs	Slow-progressor

* Based on virus loads in 90% of all samples of a given patient

** CD4+ve levels as described in the original studies

Supplementary Table: 2 Details of HIV-1 subtype, time-points of sampling, number of clones sequenced at each time point and place of study for all patients in all the three data sets							
**DATASET 1**							
**Classification in our study**	**Patient Code**	**Country**	**Subtype**	**Year of Sampling**	**Time from seroconversion months (days)**	**Accession numbers (Number of Clones)**	**Total number of Clones**
Typical-progressor	P1	United States of America	B	1983	3 (91)	AF137629-35(7)	7
			B	1984	124(426)	AF137636-45 (10)	10
			B	1984	24(730)	AF137646-54 (9)	9
			B	1985	34(1034)	AF137655-62 (8)	8
			B	1986	45 (1369)	AF137663-72 (10)	10
			B	1987	51 (1551)	AF137673-78 (6)	6
			B	1988	61 (1856)	AF137679-88 (10)	10
			B	1988	66(2008)	AF137689-96 (8)	8
			B	1988	68 (2069)	AF137697-705 (9)	9
			B	1989	77 (2342)	AF137706-15 (10)	10
			B	1989	80 (2434)	AF137716-25 (10)	10
			B	1990	87 (2647)	AF137726-35 (10)	10
			B	1990	94 (2859)	AF137736-45 (10)	10
			B	1991	98(2981)	AF137746-55 (10)	10
			B	1991	105 (3194)	AF137756-65 (10)	10
Typical-progressor	P2	United States of America	B	1983	5 (152)	AF137766-75 (10)	10

			B	1984	12 (365)	AF137776-86 (11)	11
			B	1984	20 (608)	AF137787-96 (10)	10
			B	1985	30 (913)	AF137797-807 (11)	11
			B	1986	40 (1217)	AF137808-815 (8)	8
			B	1987	51 (1551)	AF137816-823 (8)	8
			B	1988	61(1856)	AF137824-28 (5),AF137888-91(4)	9
			B	1989	68 (2069)	AF137829-32 (4) ,AF137792- 96 (5),AF137797(1)	10
			B	1989	73 (2221)	AF137833-37 (5) ,AF137878-82 (5)	10
			B	1989	80 (2434)	AF137838-43 (6) ,AF137883-84 (2)	8
			B	1990	85 (2586)	AF137844-50(7) ,AF137885 (1)	8
			B	1990	91 (2768)	AF137851-57 (7) , AF137886-87 (2)	9
			B	1991	103 (3133)	AF137858-66 (9)	9
			B	1983	126 (3833)	AF137867-77 (11)	11
Typical-progressor	P3	United States of America	B	1983	4 (122)	AF137898-907 (10)	10
			B	1983	9 (274)	AF137908-17 (10)	10
			B	1985	26(791)	AF137918-27 (10)	10
			B	1985	30(913)	AF137928-39 (12)	12
			B	1986	42(1278)	AF137940-48 (9)	9
			B	1987	55(1673)	AF137949-58 (10)	10

			B	1988	67 (2038)	AF137959-70 (12)	12
			B	1989	73 (2221)	AF137971-81 (11)	11
			B	1989	80(2434)	AF137982-94 (13)	13
			B	1990	96(2920)	AF137995-AF138003 (9)	9
Typical-progressor	P5	United States of America	B	1983	3(91)	AF138004-13 (10)	10
			B	1983	9(274)	AF138014-24 (11)	11
			B	1984	16(487)	AF138025-34 (10)	10
			B	1984	19(578)	AF138035-44 (10)	10
			B	1984	21 (639)	AF138045-54 (10)	10
			B	1985	25 (761)	AF138055-64 (10)	10
			B	1985	28 (852)	AF138065-74 (10)	10
			B	1985	34 (1034)	AF138075-84 (10)	10
			B	1986	40 (1217)	AF138085-94 (10)	10
			B	1986	42 (1278)	AF138095-105 (11)	11
			B	1986	43(1308)	AF138106-15 (10)	10
			B	1987	49(1491)	AF138116-26 (11)	11
			B	1987	56(1704)	AF138127-35 (9)	9
			B	1988	62(1886)	AF138136-45 (10)	10
			B	1988	68(2069)	AF138146-54 (9)	9

			B	1989	81(2464)	AF138155-63 (9)	9
Typical-progressor	P6	United States of America	B	1983	3 (91)	AF138166-74 (9)	9
			B	1983	9 (274)	AF138175-84 (10)	10
			B	1984	18 (548)	AF138185-94 (10)	10
			B	1984	24 (730)	AF138195-205 (11)	11
			B	1985	30 (913)	AF138206-15 (10)	10
			B	1985	36(1095)	AF138216-23 (8)	8
			B	1986	42(1278)	AF138224-30 (7)	7
			B	1986	48 (1460)	AF138231-41 (11)	11
			B	1987	54 (1643)	AF138242-52 (11)	11
			B	1989	73 (2221)	AF138253-63 (11)	11
Typical-progressor	P7	United States of America	B	1983	2 (61)	AF138305-14 (10)	10
			B	1985	25 (761)	AF138315-26 (12)	12
			B	1985	31 (943)	AF138327-37 (11)	11
			B	1986	44 (1338)	AF138338-47 (10)	10
			B	1987	50 (1521)	AF138348-61 (14)	14
			B	1987	56 (1704)	AF138362-72 (11)	11
			B	1988	62 (1886)	AF138373-82 (10)	10
			B	1989	74 (2251)	AF138383-99 (17)	17

			B	1989	80 (2434)	AF138400-11 (12)	12
Typical-progressor	P8	United States of America	B	1983	3(91)	AF138412-18 (7)	7
			B	1983	10 (304)	AF138419-28 (10)	10
			B	1984	17 (517)	AF138429-36 (8)	8
			B	1985	29 (882)	AF138437-44 (8)	8
			B	1985	35 (1065)	AF138445-52 (8)	8
			B	1986	41(1247)	AF138453-60 (8)	8
			B	1986	46 (1399)	AF138461-69 (9)	9
			B	1987	53 (1612)	AF138470-77 (8)	8
			B	1987	59 (1795)	AF138478-86 (9)	9
			B	1988	65 (1977)	AF138487-96 (10)	10
			B	1988	70 (2129)	AF138497-506 (10)	10
			B	1989	81 (2464)	AF138507-18 (12)	12
			B	1990	95 (2890)	AF138519-30 (12)	12
Slow-progressor	P9	United States of America	B	1983	3(91)	AF138531-40 (10)	10
			B	1983	9 (274)	AF138541-49 (9)	9
			B	1984	17 (517)	AF138550-58 (9)	9
			B	1986	39 (1186)	AF138559-68 (10)	10
			B	1988	63 (1916)	AF138569-80 (12)	12

			B	1990	86 (2616)	AF138581-90 (10)	10
			B	1991	104(3164)	AF13891-96 (6),AF138644-46(3)	9
			B	1992	111(3377)	AF138597-604 (8),AF138647-48(2)	10
			B	1992	117 (3559)	AF138605-12 (8),AF138649-50 (2)	10
			B	1993	122 (3711)	AF138613-22 (10)	10
			B	1993	129 (3924)	AF138623-33 (11),AF138651(1)	12
			B	1994	134 (4076)	AF138634-43 (10)	10
Slow-progressor	P11	United States of America	B	1983	3(91)	AF138652-65 (14)	14
			B	1985	29 (882)	AF138666-73 (8)	8
			B	1986	42 (1278)	AF138674-79 (6)	6
			B	1987	58 (1764)	AF138680-89 (10)	10
			B	1988	70 (2129)	AF138690-96 (7)	7
			B	1991	100 (3042)	AF138697-703 (7)	7
						Total	1032
**DATASET 2**							
Typical-progressor	A	Italy	B	Not available	365	AF105432 -AF105445(14), AF105717-AF105729(13)	27
			B		1095	AF105446-AF105460(15), AF105730-AF105744(15)	30
			B		1460	AF105461-AF105473(13), AF105745-AF105759(15)	28

Typical-progressor	B	Italy	B	Not available	2555	AF105474-AF105488(15) , AF105760-AF105774(15)	30
			B		3285	AF105489-502 (14) , AF105775-AF105789(15)	29
			B		4015	AF105503-AF105517(15), AF105790-AF105804(15)	30
Typical-progressor	C	Italy	B	Not available	2373	AF105518-AF105530(13), AF105805-AF105820(16)	29
			B		2378	AF105531- AF105544(14), AF105821-AF105835(15)	29
			B		3103	AF105545- AF105558(14), AF105836-AF105845(10)	24
Slow-progressor	D	Italy	B	Not available	2920	AF105559-AF105573(15), AF105846-AF105858(13)	28
			B		3285	AF105874-AF105886(13), AF105859-AF105873(15)	28
			B		3650	AF105587- AF105594(8), AF105874-AF105887(14)	22
Slow-progressor	E	Italy	B	Not available	2738	AF105595- AF105609(15), AF105888-AF105902(15)	30
			B		3103	AF105610-AF105624(15), AF105903-AF105914(12)	27
			B		3650	AF105625-AF105637(13), AF105915-AF105927(13)	26
Slow-progressor	F	Italy	B	Not available	3833	AF105638- AF105652(15), AF105928-AF105939(12)	27
			B		4198	AF105653- AF105666(14), AF105940-AF105947(8)	22
			B		4563	AF105667- AF105680(14), AF105948-AF105961(14)	28
						Total	494
**DATASET 3**							
Typical-progressor	59	SPAIN	B	1998	731	AY498227- AY498246(20)	20
			B	2000	1278	AY498247- AY498266(20)	20

			B	2001	1826	AY498267- AY498286(20)	20
			B	2004	3105	EF118762- EF118786 (25)	25
Typical-progressor	60	SPAIN	B	1999	1826	AY498287- AY498306(20)	20
			B	2000	2192	AY498307- AY498326(20)	20
			B	2001	2557	AY498327-AY498346(20)	20
			B	2002	3105	EF118787- EF118806 (20)	20
Slow-progressor	30	SPAIN	B	1998	3287	AY497931-Y497947(17),AY497954-AY497955(2)	19
			B	1999	3653	AY497989-AY498008(20)	20
			B	1999	4200	AY498032-AY498043(12),AY498048-AY498055(8)	20
			B	2001	4566	AY497948-AY497949 (2),AY497956-AY497973 (18)	20
			B	1999	4748	AY497950- AY497953 (4),AY497974-AY497988 (15)	19
			B	2002	4931	AY498044- AY498047(4),AY498056-AY498070 (15)	19
						Total	282
